# Exploring the Education and Perceptions of Dental Professionals Toward Poverty and Oral Health Disparities: A Scoping Review

**DOI:** 10.1111/cdoe.70039

**Published:** 2025-11-11

**Authors:** Lisa Allen, Janine Doughty, Samantha Beattie, Charlotte Hardman, Sondos Albadri

**Affiliations:** ^1^ NIHR Primary Dental Care School of Dentistry, University of Liverpool Liverpool UK; ^2^ School of Dentistry University of Liverpool Liverpool UK; ^3^ Department of Psychology University of Liverpool Liverpool UK

## Abstract

**Objective:**

The objective of the present study was to map the available research, investigating how dental professionals perceive those who experience poverty, and what educational interventions are available in this area, with the aim of providing a narrative summary and identifying key research gaps within the literature.

**Method:**

Following Joanna Briggs scoping review methodology, databases searched included PubMed, Scopus, CINAHL, Dentistry and Oral Sciences, Health Research Premium and Web of Science databases. A manual search was also performed on Google Scholar to identify grey literature. Search strategies included medical subject headings and key terms including *poverty*, *financial hardship*, *social deprivation*, *oral health*, *oral diseases*, *dental caries*, *communication*, *learning and dental education.*

**Results:**

A total of 1046 articles were assessed for eligibility, of which 34 studies met the predefined inclusion criteria. The studies included in this review highlight conflicting understandings about what it means to be living in poverty. Outreach activities shared lived experiences, and immersive opportunities were reported to improve dental professionals' attitudes and willingness to provide dental care for people living in poverty.

**Conclusion:**

To the study teams' knowledge, this is the first review to examine specifically how poverty and oral health are understood by the dental profession. This review highlights the need for further research as to the long‐term effectiveness and cost‐effectiveness of educational interventions to increase understanding.

## 1 Introduction

Poverty is a global public health issue. A person living in poverty lacks “resources to obtain the type of diet, participate in activities and have the living conditions and amenities which are customary, or at least widely encouraged in the societies in which they belong” [1]. In 2015, world leaders made a commitment to end poverty in all its forms everywhere [2]. Despite this commitment, there has been a surge in poverty rates globally with around 700 million people living in extreme poverty [3].

The WHO Oral Health Report 2022 highlighted associations between changing chronic disease patterns and socioenvironmental determinants such as the strong associations between poor oral health and poverty [4]. Epidemiological evidence consistently associates social gradients with the prevalence of tooth decay, tooth loss, the occurrence, severity and mortality of oral cancer, self‐rated oral health, oral hygiene‐related quality of life, oral hygiene and service use [5, 6, 7, 8]; with children living in the most deprived areas of England being almost 3 times as likely to have experience of dentinal decay than those living in the least deprived [9]. Despite the ubiquity of evidence linking poverty and oral health, there is limited research available on current perceptions of dental teams and their education and training; to understand the challenges of living in poverty, effectively address the needs of patients and provide patient‐centered care. In other areas of healthcare such as with cancer therapy [10] this approach has improved the quality of life of patients and reduced healthcare spending [10]. Therefore, this scoping review aimed to present what is known about how dental professionals perceive people living in poverty, and what educational interventions have been utilized to teach dental teams about poverty and oral health.

## 2 Methods

The reporting of this scoping review follows the Preferred Reporting Items for Systematic Reviews and Meta‐Analyses extension for Scoping Reviews (PRISMA‐ScR) [11] checklist (S1) and its methodology is based on the Joanna Briggs Institute (JBI) framework [12]. A scoping review was chosen to determine the nature and extent of published literature in this area. All members of the research team were involved in protocol development (https://osf.io/hg2aj/).

### 2.1 Patient and Public Involvement

NIHR INVOLVE define public involvement as “research being carried out ‘with’ or ‘by’ members of the public rather than ‘to’, ‘about’ or ‘for’ them” [13]. As part of a larger research project, a patient and public group (PPI) was formed prior to the search, consisting of a group of people with lived experience of poverty; as identified by their attendance at a community pantry; an organisation, whereby food and other essential items are sold at a reduced cost, often to individuals experiencing financial hardship. It is believed that up to 95% of people referred to food banks live in destitution, meaning that they cannot afford necessities like food and shelter [14]. As such the study group determined the importance of seeking their views. Members were invited to review the research questions to ensure they were relevant and important, the salience of key terms and identified priority areas.

### 2.2 Inclusion and Exclusion Criteria

The inclusion and exclusion criteria are listed in Table 1 and derived from the consideration of participants, concept, and context [12]. This enabled the study team to identify key topic themes to include in our search strategy. An open search was conducted to capture relevant data.

**TABLE 1:** Inclusion and exclusion criteria.

Criteria	Inclusion	Exclusion
Date	Open search conducted with no start date until 31st May 2025	
Language	English	Other languages
Country	Any (provided the paper is written in English)	
Type of study	Case reports, qualitative studies, cross sectional studies, case–control studies, cohort studies., summary reports commentaries, dissertations and conference extracts	
Concept	Poverty including additional synonyms, social deprivation and financial hardship	Topics that may include but are not specific to poverty as defined in this study e.g., broad reference to social determinants of health
Context	Any papers pertaining to perception of dental poverty and how well dental teams communicate with their patients from low‐income groups. Papers also focusing on means of educating dental teams surrounding poverty including dental education, learning and how the concept of poverty is communicated to dental professionals	Education of other health professionals
Population	Dental professionals including dentists, dental students, dental hygienists, oral hygienists, dental therapists, dental nurses and dental receptionists working in any dental setting	Other health professionals

### 2.3 Study Identification

With the assistance of a University of Liverpool‐based librarian who provided support to identify key words, index terms and appropriate database selection, a search to find relevant studies (S2) was undertaken on PubMed, Scopus, CINAHL, Dentistry and Oral Sciences, Health Research Premium, Web of Science electronic databases and Google Scholar to identify grey literature. Studies were exported into Rayaan reference manager and duplicates were removed. Two independent reviewers (LA, SB) carried out a pilot search of two online databases (PubMed and Scopus) screening 20 records to ensure standardisation of text words found in the title and abstract of the retrieved papers. Thereafter, a refined search strategy was enacted for each database.

Searches were undertaken in May–July 2023 and updated in May 2025.

### 2.4 Evidence Screening

The screening and selection procedure was applied using the PRISMA‐ScR flowchart [11].

Inclusion criteria were applied independently by LA and SB to all abstracts. Full texts for inclusion were identified. Conflicts were resolved by meeting and discussing until a consensus was reached.

Finally, the reference list of identified reports and articles was searched for additional sources. Additionally, review articles (e.g., Systematic reviews and Scoping reviews) were identified for citation sourcing.

The search strategy for PubMed is presented in Table S3.

### 2.5 Charting the Data

Following screening, relevant literature was transferred to Microsoft Excel and reviewed by team members LA, SB to include bibliographic information: year of publication, author, title, journal, geographic location, study information, design, sample size (where applicable), study aims and key findings. Qualitative and quantitative data were extracted from each document as it was important to observe participant perceptions and experiences regarding their educational experiences. Data were extracted by one reviewer and verified by another. Discrepancies were resolved via discussion and if a consensus was not reached a third reviewer made the final decision.

### 2.6 Data Summary and Report

A thematic analysis was undertaken with each of the included papers being read in detail and key findings summarised into themes based on similar content, then placed under subheadings to reflect these themes [15]. This was undertaken by LA and SB using a bottom‐up, data‐driven approach who met to discuss the process. If there was disagreement, the authors would meet and discuss until a consensus was reached. No critical appraisal was undertaken as it is not required for the purpose of a scoping review [12].

## 3 Results

**FIGURE 1:**
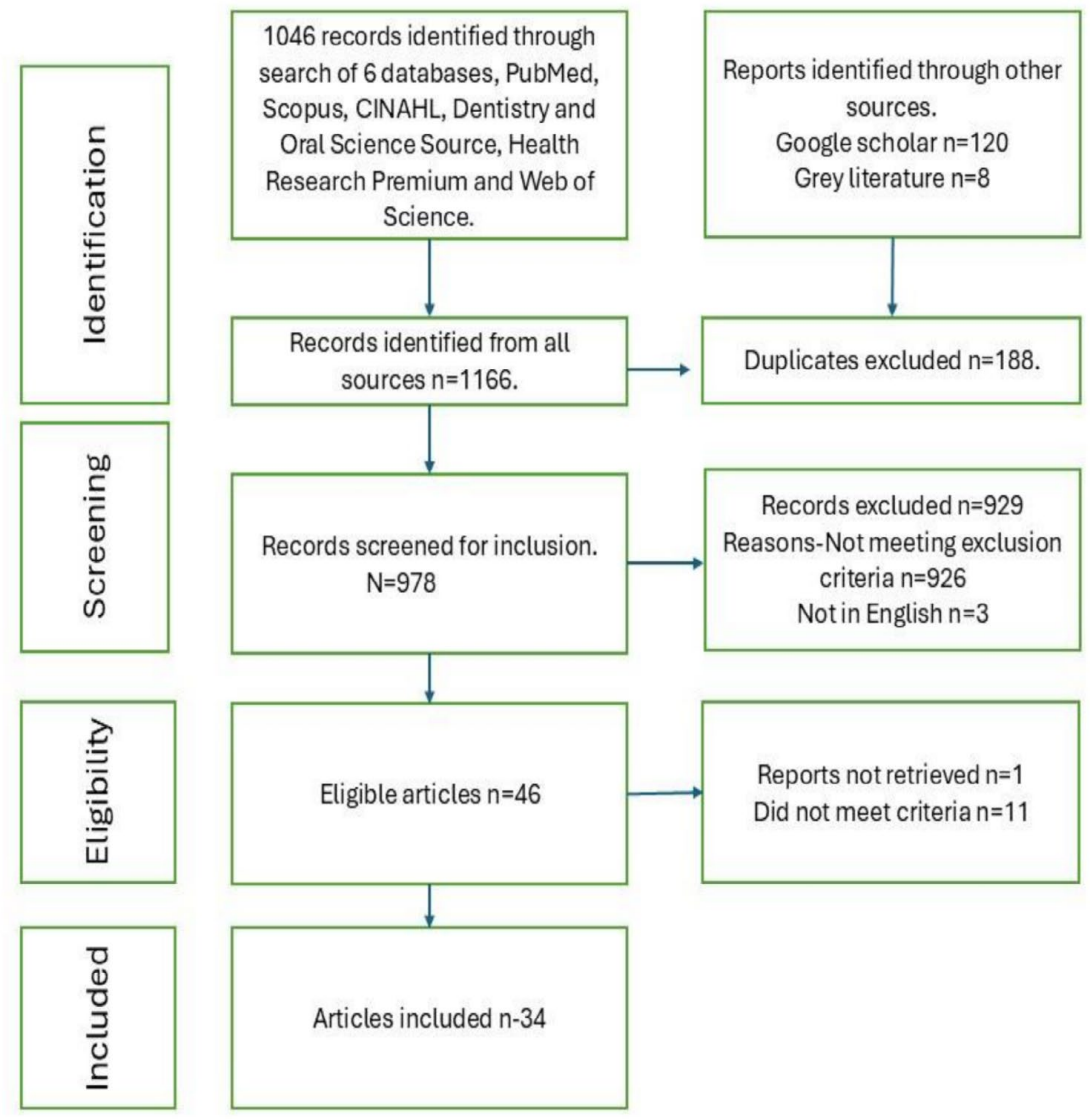
PRISMA flowchart of study selection.

The search process and outcomes are presented in Figure 1.

### 3.1 Characteristics of Included Studies

Of the 34 articles, there were 10 quantitative studies (29.4%), 15 qualitative studies (44.1%), two mixed methods studies (5.9%), five summary reports (14.7%), one conference report (2.9%), and one educational case study (2.9%).

Sources were published between 2004 and 2024 (Table S4). Most articles (*n* = 19) were authored within the United States of America and nine in Canada. Others were from the UK, Brazil, Peru, Australia, and East Africa. Most articles were published in the Journal of Dental Education.

### 3.2 RQ 1: How Do Dental Professionals Perceive Patients Who Live in Poverty?

The views of dental professionals generally differed based on their experience working with patients living in poverty in all of the professional groups, with studies exploring the views of dentists, periodontists, dental hygienists, dental assistants and dental students. Studies described how their divergent opinions of victim blaming vs. professional responsibility and how dental professionals' lived experiences, both personal and professional, shaped their perspectives of people living in poverty. The following section presents these two themes.

#### 3.2.1 Dichotomous Perceptions of People Living in Poverty

Some dentists did not express awareness or knowledge regarding the competing needs of underserved patients [16] with poverty considered a distant subject for dental professionals and students, a responsibility of the government or of poor individuals themselves [16, 17]. Additionally, among dental students, there was a lack of outrage that patients often had to resort to drastic measures for their care, demonstrating a sense of powerlessness [18].

Dental professionals and dental students from Canada [18] and Brazil [19] demonstrated two contrasting perspectives about people on social assistance:

1. Individualistic deficit perspective: This perspective explains poverty as a result of individual failures rather than broader social or economic conditions. In this view, individuals may be blamed for their poverty with a focus on personal attributes such as a lack of capabilities, motivation and poor decision making. For example, one participant highlighted personal choice as being responsible for living in poverty: “It's like they don't want to get themselves out of the situation” [18].
2. Socio‐ life course perspective: Students with this perspective described structural rather than individual causes of poverty, recognising that life experience is shaped by various social conditions. Dental professionals with this perspective displayed more empathy toward people living in poverty; highlighting that “lack of education and no encouragement from their parents” or other circumstances such as “car accidents, work injuries or ill health” may prevent improvement in circumstances [18].

Similarly, dental hygienists who reported experience in community service and higher job satisfaction exhibited more empathy toward people living in poverty [20, 21]; and a study including periodontists also indicated that attitudes were more positive toward “pro bono” and Medicaid patients depending on their educational experiences [22].

#### 3.2.2 Personal and Professional Experiences

Though many students were willing to provide care for people living in poverty, others demonstrated a reducing willingness as they progressed through dental school [23]. The authors interpreted that this was related to the loss of idealism and increasing perceptions of the reality of dental practice [24]. Although patients frequently faced systemic obstacles to accessing care, dental teams often pointed to the broader consequences of these barriers—such as low reimbursement rates and high rates of missed appointments—as reasons for their perspectives [18].

In contrast, dental professionals working in areas of high deprivation showed greater understanding of patients' social context, taking time, and showing empathy, avoiding moralistic attitudes, overcoming social distances, and favouring direct contact with patients [25]. Studies proposed that different models of care were required to meet the needs of vulnerable populations [18]. Two studies [26, 27] demonstrated that engagement can be encouraged through public health initiatives, in areas where access to dental care is difficult by encouraging a greater level of volunteerism [26] and advancing the oral health workforce and training [27].

### 3.3 RQ 2: How Are Dental Professionals Educated About the Relationship Between Poverty and Oral Health?

Educational interventions spanned undergraduate education, outreach activity and continued professional education of qualified dental professionals. Each of these is described as a separate theme and detailed in the following paragraphs.

#### 3.3.1 Undergraduate Training

Overarchingly, lectures provided the factual basis to raise consciousness along with small group discussions that encouraged “cross validation.” Accessing students earlier in their training was reported to enhance their understanding of social theories and careful curriculum design was essential to prevent disillusionment and maintain engagement [28]. Education about Medicaid patients was directly related to supportive professional attitudes and communication skills with low oral health literacy patients [29].

In addition, simulation can be effective in improving student attitudes toward poverty [30, 31, 32] and enabling those with lived experience to share their stories [33].

Community‐based education and reflective learning were also advocated to support dental students in developing professional values [34, 35]. Practicing or living in low‐income settings or rural communities shows promise for improving proficiency in managing community oral health problems and enhances preparedness for treating underserved populations [36, 37, 38, 39, 40, 41, 42]. The use of social histories in an outreach setting can be used as a vehicle to study the social determinants of health and avoid stereotyping the local population [43].

Opportunities for experiential learning can improve students' understanding of social obligations, cultural humility, social awareness and awareness of healthcare systems, commitment to service, personal and professional growth, thus leading dental students to make morally inclusive choices [42]. Community‐based dental education may have the potential to positively influence dental students' likelihood of selecting a community dental clinic as a first career choice [37]. However, outreach programmes face challenges such as lack of continuity and comprehensiveness of care and students lack prior knowledge of dental services within the welfare programme [24].

It was suggested that dental practitioners [38] need to have a better understanding of poverty and learn from medicine to implement programmes that culture and cultivate where staff, faculty and practitioners work toward the elimination of stereotyping with interdisciplinary training advocated [44].

#### 3.3.2 Postgraduate Training

There were very few studies that presented educational strategies in postgraduate education. Those that did, focused on people living in poverty sharing their stories with dental professionals. People described their lives, including the importance of loss of teeth and stigma, the importance of empathy, barriers to accessing dental services and everyday life on welfare [37, 45, 46, 47]. After engaging with video recordings, dental team members described a newfound understanding of poverty and the nature of life on welfare [39]. Other educational courses included community health and advocacy training (CHAT) among paediatric dental residents [48] who reported a greater perception of disease management and risk assessment tools when working with low‐income families. Two studies [47, 49] described the training for the dental team in counselling skills and how to approach sensitive conversations such as financial screening [49].

## 4 Discussion

This scoping review aimed to determine perceptions of dental professionals and their education surrounding patients experiencing poverty by evaluating research articles published prior to June 2025. Findings indicate that dental professionals' and students' understanding of poverty is limited and therefore highlight the potential for educational strategies to improve engagement and competency with patients experiencing poverty. A correlation between experience with underserved patients and beliefs was also apparent.

Dichotomous perspectives about people living in poverty were found throughout the studies dictating whether dental professionals tended toward victim‐blaming or empathetic views with some professionals demonstrating a clearer understanding of poverty and the challenges associated with providing care.

Despite dental students expressing a willingness to treat people experiencing poverty during their initial training this sentiment appeared to diminish as they came to understand the realities of dental practice. Consistent with medical literature, efficiency required in dental practice can be at odds with providing human‐centred, empathetic care and the challenges of real‐world dentistry may prevent the enactment of idealistic practices of caring for dental patients experiencing poverty [50].

In addition, studies describe intrinsic characteristics of dental professionals that can determine professionals' willingness to engage with underserved populations; those characterised as altruistic carers show tendencies toward caring for exceptionally deserving patients [51]. Dental professionals with these characteristics may demonstrate more empathy toward people living in poverty, be more engaged with educational programmes and be more willing to work in areas with higher deprivation. Altruistic carers are more commonly found to be employed in Community Dental Service settings providing dental care for underserved populations.

Recruitment to dentistry from minority or underrepresented groups will also contribute to creating a diverse range of culturally appropriate practice and care toward patients from dental professionals who understand the nuances of their lived experience [52]. It is therefore important to consider in the first instance how dental professionals are recruited to ensure diversity and understanding.

This scoping review identified several educational interventions that aimed to improve dental professionals' understanding of poverty. Whilst some studies presented lectures and critical discussion, others explored the value of simulation and reflection to deepen consciousness. Critical reflection and validity testing of underlying beliefs and judgments may lead to new ways of posing a problem and ultimately restructure beliefs [53]. Reflection therefore can be a means to transformative learning.

Other studies have highlighted that even after training, dental professionals struggle with having difficult conversations about stigmatising or taboo topics, highlighting the importance of immersive approaches such as outreach experiences that fundamentally change cultural competency as opposed to didactic teaching approaches [54].

Poverty is rarely experienced as an isolated phenomenon; it is commonly coupled with multiple, interconnected social issues known as the social determinants of health. Therefore, the skills of the dental practitioner move beyond empathy and require dental professionals to be competent in having difficult conversations about sensitive topics such as unemployment, domestic abuse and the criminal justice system.

Current dental educational practices continue to be dominated by cause‐effect teaching rather than the social determinants of health [55] and struggle to integrate socio‐cultural training [56]. There is also an emphasis on knowledge translation which is objective, impersonal and separate from those it is intended for [57] with little guidance as to how soft skills can be assessed with validity. Health professionals who believe deeply and compassionately about patient care can role model empathy and engage in ongoing discussions for improving the care of patients living in poverty. Calling attention to what they are modeling can encourage a “pedagogy of discomfort” in which students are encouraged to scrutinise their ideals [58].

Studies in this scoping review highlighted the importance of immersion in underserved communities as a means to enhance empathy and cultural sensitivity. An example of immersive learning within the wider medical field is the development of “deep end” GPs [59] in which they have collaborated in areas of deprivation to focus on training needs required to treat the complexities of patients living in poverty, and to explore approaches to incentivising practice and cultivating empathetic care. Such a scheme would better prepare dental professionals to provide “deep‐end dentistry” [60].

Sharing patient narratives of lived experience showed promise for improving the dental team's understanding of life and health trajectories. Service user action research allows for those with lived experience to contribute to and improve services [61]. Thereby both parties come to an understanding of principles that govern their interactions, rather than experiencing unilateral physician decision making [62]. Studies in this area are worthy of further exploration.

### 4.1 Recommendations for Research and Practice

Future research areas include studying the longitudinal impact of educational interventions and the cost‐effectiveness of such programmes. Service user action research should be developed to allow for those with lived experience to contribute to and improve services. Continued collaboration with wider networks such as the development of “deep end dentistry” programmes is also advocated.

### 4.2 Study Limitations

There were some limitations to the study; for example, most included studies were summary reports or cross‐sectional designs, with one longitudinal study and no randomised controlled trials identified. While a scoping review is a useful aid to map literature on a broad topic and form the basis for future enquiry, it does not critically analyse the literature. Therefore, the findings cannot be generalised and do not aim to measure the effectiveness of interventions.

## 5 Conclusion

In conclusion, this is the first study to bring together peer‐reviewed literature demonstrating how the dental profession perceives and is educated about poverty. The studies included in this review highlight conflicting understandings about what it means to be living in poverty. Outreach activities sharing lived experiences, and immersive opportunities were reported to improve dental professionals' attitudes and willingness to provide dental care for people living in poverty, but high‐quality longitudinal studies are required to understand the long‐term impact and cost‐effectiveness.

## Ethics Statement

The authors have nothing to report.

## Conflicts of Interest

C.A.H. has been Co‐Investigator on a research grant funded by the American Beverage Association (paid to institution). They currently receive honoraria for their role on the Global Nutrition Advisory Council for Mars (paid to institution) and are primary supervisor on a PhD studentship funded by Coca‐Cola. They report personal fees for their role on the UK Government's Food Standards Agency's Advisory Committee on Social Sciences, and they have previously received personal honoraria from the International Sweeteners Association and the International Food Information Council. They report an unpaid role as a trustee of Feeding Liverpool. All are for work unrelated to the submitted manuscript.

## Supporting information


**Data S1:** cdoe70039‐sup‐0001‐Supinfo1.pdf.

**Appendix S1:** cdoe70039‐sup‐0002‐AppendixS1.docx.

**Appendix S2:** cdoe70039‐sup‐0003‐AppendixS2.docx.

## Data Availability

The data that support the findings of this study are available from the corresponding author upon reasonable request.
