# Supplementary material for: Exploring the Education and Perceptions of Dental Professionals Toward Poverty and Oral Health Disparities: A Scoping Review
Source: Community Dent Oral Epidemiol. 2025 Nov 11;54(1):22–9. doi: 10.1111/cdoe.70039 (PMC12808863; doi:10.1111/cdoe.70039)
Supplement: Supplementary file 3 — Appendix S2: cdoe70039‐sup‐0003‐AppendixS2.docx. [file CDOE-54-22-s002.docx]

**14: Appendices**

**S1 PRISMA-SCR Checklist see Appendix S1**

**Appendix S2: Search strategy- A full search strategy from the PubMed included 31^st^ May 2025**

| # | Searches | Number |
| --- | --- | --- |
| 1 | Poverty  Plus, additional synonyms found | 81,309 |
| 2 | Social deprivation | 282 |
| 3 | Financial hardship | 1777 |
| 4 | 1 or 2 or 3 | 85,286 |
| 5 | Oral Health | 76,089 |
| 6 | Oral diseases | 26,426 |
| 7 | Dental caries | 61,308 |
| 8 | 5 or 6 or 7 | 145,238 |
| 9 | Communication | 901,304 |
| 10 | Learning | 1118,306 |
| 11 | Dental education | 27,314 |
| 12 | 9 or 10 or 11 | 1938,340 |
| 13 | 4 AND 8 AND 12 | 144 |
| 14 | Limit 13 to English Language | 143 |
|  |  |  |

**Appendix S3**

PubMed, Scopus Poverty (MeSH) OR Financial hardship

OR Social deprivation.

AND Oral disease OR Dental caries OR Oral health (MeSH)

AND Dental education OR Communication OR Learning.

----------------------------------------------------------------------------------------------------------------

**Data extraction form for PUBMED search.**

**Table S4-Data extraction table.**

| **Study** | **Location** | **Study design** | **Sample size (if applicable)** | **Aim** | **Key findings** |
| --- | --- | --- | --- | --- | --- |
| Treadwell, Catalanotto, Warren et Al 2017 (16) | United States | Qualitative study  Focus groups | 16 (15 dentists and one dental hygienist) | Elicit dental practitioners’ perceptions of the barriers of underserved and unserved patients seeking oral health care. | Participants did not express awareness or knowledge of competing needs of underserved patients. |
| Reis, Rodriguez, Macauley et Al 2014 (17) | Canada | Cross sectional qualitative study-semi structured interviews | 12 dental students | To understand dental students’ perceptions of an attitudes towards poverty and dental care provided to those living in poverty. | Poverty considered a distant subject and responsibility of the government themselves. Suggests a need to increase critical consciousness about oral health inequalities. |
| Loignon, Landry, Allison, 2012 (18) | Canada | Qualitative Retrospective study based on in depth interviews. | 53 dentists | To understand how dentists perceive poverty and people on social assistance. | Patients often feel misunderstood or stigmatized.  Study revealed two perspectives 1: Individualistic-deficit perspective: Predominant perspective, poverty explained by individual factors-emphasized individual negative attitude towards work and lack of capabilities. 2: Socio-life course perspective-described as a structural rather than individual process and therefore displayed more empathy. |
| Santos, Madathil, Zuanon et Al 2017 (19) | Brazil | Cross sectional quantitative survey. | 766 students | To investigate dental students’ attitudes towards people living in poverty and the extent to which their perceptions were associated with their willingness to treat those people in the future. | Positive attitudes found towards those living in poverty however a high proportion (35%) reported thinking they were different from the rest of the population. Dental students’ perception of poverty linked with their willingness to treat underserved population in the future. |
| Marsh 2012  (20) | United States | Cross sectional quantitative survey. | 109 registered dental hygienists. | To assess dental hygienist attitude towards caring for the underserved. | Discriminant analysis indicated a strong correlation among registered dental hygienists community service and job satisfaction. |
| Cobb 2019  (21) | United States | Cross sectional qualitative study | 55 dental hygienists | To evaluate perceptions of dental hygienists who work in underserved communities. | Providing dental care to patients with an empathetic and collaborative spirit is critical. |
| Garfinkle, Richards, Inglehart 2010  (22) | United states | Cross sectional quantitative study | 291 periodontologists and 64 periodontal residents | To explore how well the dental education of periodontists prepared them to treat underserved patients, to assess professionals attitudes and confidence when treating these patients as well as professional behaviours and whether educational experiences are related with attitudes, confidence and behaviours. | Students were more confident treating patients on Medicaid and pro bono cases than practicing clinicians. The quality of predoctoral and graduate educations regarding underserved patients correlates with attitudes, confidence and behaviours of providers concerning providing care for these patients. |
| Flynn, Luthra, Blue 2017  (23). | United states | Longitudinal quantitative study. | Second year students 97 (98.9%) and fourth year students 67 (68.3%) | To determine whether intraprofessional education with dental therapy students would affect dental students attitude towards caring for underserved populations. | Dental students had positive attitudes towards treating vulnerable patients on entering dental school, but their attitudes changed became less positive over four years regardless of intra professional training with dental therapy students. |
| Reis 2012 (24) | Canada | Qualitative  Case study | 12 dental students. | To examine in-depth the perceptions and attitudes of final year dental students at McGill University towards poverty and the dental care provided to low-income patients. Secondary objectives: (i) To explore the extent to which students feel that their education in dentistry has prepared them to work with low-income patients; (ii) To understand if these perceptions shape students' plan for their professional careers. | Dental students perceived poverty as a distant subject and as a responsibility of the government or of poor individuals themselves. Students identified challenges of outreach programme such as lack of continuity and comprehensiveness of care as well as deficient compliance with clinical guidelines and lacked knowledge of dental services within the welfare programme. |
| Loignon, Allison, Landry, Et Al 2010. (25) | Canada | Qualitative research based on semi-structured interviews. | 8 dentists | To identify specific approaches and skills identified by dentists for more effective treatment of people living in poverty and addressing their needs. | Five-faceted socio-humanistic approach that involved: (1) understanding patients' social context; (2) taking time and showing empathy; (3) avoiding moralistic attitudes; (4) overcoming social distances; and (5) favouring direct contact with patients. |
| Dubay; Parker; DeFriese, 2005 (26). | United states | Summary report | NA | Outlines strategies to improve access to low-income population within North Carolina. | Strategy one- Increased access to low-income patients.  Strategy two- Increasing supply of dental professionals.  Strategy 3- Increasing dental care available to special needs populations.  Strategy 4- Increasing public awareness of the importance of oral healthcare.  Encourage a greater level of volunteerism among dental care professionals to serve the needs of the local population. |
| Leake,2005 (27) | Canada | Conference report following presentation at access and care symposium. | NA | An overview of presentations from agencies that train dental care providers to meet the needs of Canadians with restricted access to care | Highlighted frustrations with current trends and apparent indifference of policymakers and presented some success in alternative models of care. Also, reduction in healthcare funding to local dental school resulting in “steady decline in the availability of care for vulnerable populations,” |
| Habibian, Seirawan, Mulligan 2011  (28). | United states | Cross sectional  Quantitative study. | 146 | Students’ attitudes measured across 4 years of dental school relating to societal expectations, dentist/student responsibility, personal efficacy, and access to care. | -This research showed that dental students’ attitudes towards treating underserved patients declined as they progressed through dental school. -Suggests that this might be due to reducing idealism and increased understanding of complexities of practice life |
| Albino, Inglehart, Tedesco, 2012 (29). | United states | Summary report | NA | 1: To describe the oral health related situation of the population groups who experience disparities in oral health. 2: Discuss ways in which dental education can optimally address these issues in the future. 3: Conclude with reflection on the past and future contributions of the JDE. | Discussed challenges to access care in low-income groups, special care patients and children. In relation to low-income groups suggested reduced access to Medicaid programmes as a result of funding issues and increased failure of appointments challenging. Findings indicate education about Medicaid patients relates to professional attitudes and behaviour and that communication skills can support patients with reduced oral health literacy. Educational programmes in community-based settings supported. |
| Sams, Lampiris, White et Al 2019 (30) | United States | Cross sectional quantitative survey post exercise and following community rotation. | 34 2^nd^ year dental hygiene students and 23 dental assisting students. | Evaluation of a poverty simulation exercise on students to understand daily challenges faced by low-income families. | The majority of students felt that the exercise was useful in preparing them to understand the challenges, indicating that they were less judgemental and more empathetic. |
| McKenzie, Tilashalski, Abou-Arraj, Et Al 2019. (31) | United states | Multiple cohort study, mixed methods survey. | 497 Second- and Fourth-year dental students and 12 Periodontic residents. | To investigate how multiple cohorts of dental students evaluated simulations utilising standard patients and manakins and to explore evaluation of a simulation that combined social determinants of health with oral health education. | Students viewed simulation positively on the whole but perceived conversations with low-income patients more negatively. Request for educational techniques and resources to support those on low income. |
| Lampiris, White, Sama et Al 2017.  (32) | United states | Cross sectional quantitative study- Retrospective Pretest and postest given. | 69 | To determine if dental students understanding of daily challenges faced by families of low income changed as a result of poverty simulation. | Use of 21-item attitude toward poverty scale.  Poverty simulation is effective in raising dental students’ awareness of challenges faced by low-income families.  Attitudes influenced by facilitator explanation. |
| Holden, Leadbeatter, 2021 (33) | Australia (Sydney) | Qualitative study analysing reflective statements | 92 students  (45% from Canada). | Explores how the learning experience of first year dental students have impacted on their conceptualisation of the social determinants of health. | Core themes- Professional attitudes, structural barriers, the social gulf, learning about SDM.  Coming to an understanding of the social determinants of health requires sustained attention to social theories, practical experiences as well as institutional attitudes that could be achieved through an intentional curriculum design. |
| Graham, 2006 (34). | United states | Summary report | NA | To describe how dental educators educate dental students about oral health care disparities. | Admission selection- Attempt by dental schools to admit a proportion of students from underrepresented minority population groups.  Educational methods  Lectures- Helpful in providing factual basis for raising consciousness of students regarding issues.  Small group discussions-Facilitates exploration of beliefs by “cross validation” interactions.  Community based Health Education.  Reflective learning- Seems to be effective in helping students embrace professional values.  Dental school-based clinics, community-based clinics, continuing education. |
| Sager, Blue 2019 (35). | United States | Qualitative study | 83 dental students and 18 dental hygienists | To explore the meaning of community-based experiences for senior dental and dental hygiene students through the use of critical incident essays. | The main themes arising from the study were professional responsibility, a willingness to volunteer, and an understanding of the importance of oral health education. Results suggest that community-based learning, with reflection, challenged how students perceived oral health disparities. |
| Bernabe, Bernal, Neira 2006 (36). | Peru | Qualitative assessment of Community based dental education curriculum. | NA | To discuss the teaching-learning experiences in dental public health at the undergraduate level; in order for the student to become capable in recognising community socioeconomic-cultural conditions, so as to understand multiple causes of general and oral health problems. | Two well defined stages: 1: Experiences in low-income urban communities- consists of 5 components of social dentistry and 2: Experiences in low-income rural communities.  Time spent living in community.  Shows students reach a good level of proficiency in the management of community oral health problems. |
| Levesque, Levine, Bedos, 2015. (37) | Canada | Qualitative case study, using semi structured interviews. Digital recorded. | 15 dental team members. | Onsite course that aims to elicit effective learning and critical reflection on practises, through the use of educational integrating patient narratives and a short film. | Barriers to humanizing care:  1: Belief in the ineluctable commoditization of dentistry. 2: “Equal treatment” a belief constraining concern for equity and recognition of discriminatory practises.” 3: Biomedical orientation to care. 4: Stereotyping of patients into “deserving” and “non deserving” poor.  Require multi-level and multi sectorial action if gains in social equity to be made. |
| Piotrowski, Stenafac, Fitzgerald, Et Al 2012. (38) | United states | Qualitative/Quantitative survey pre and post Community based dental rotation.  Quantitative analysis of career choice compared to pre rotation and number of weeks in rotation. | 58 students (randomly chosen half of 4^th^ year students) | To study the influence of community based dental education (CBDE) on dental students perceived ability to treat underserved patients before and their selection of community dental clinics as a first career path. | Data suggests that several weeks spent in Community rotations- specifically more than 5 weeks can increase the likelihood that a student will select a community dental clinic as a first career choice |
| Shah, Dempster, Singhal Et Al 2023 (39) | Canada | Cross sectional qualitative survey. | 221 | Explores dental students’ comprehension of dentistry’s social contract using the concept of moral inclusion, moral community, and empathy. | The study found that students made morally inclusive choices which implied that they had a basic understanding of social obligations.  Evidence suggests working in a rural community can strengthen preparedness to treat underserved populations |
| Bomfim, Della Bona, Cury Et Al 2024. (40) | Brazil | Summary report | NA | To report the challenges for training and practice for the Brazilian primary dental caries in a universal health system. | Considering the WHO agenda, Brazil needs to advance the innovative oral health workforce, the integration of oral health into primary care, the population access to essential dental medicines and optimal fluorides for caries control. |
| Holtzman, Seirawan 2009 (41). | United states | Longitudinal quantitative study. | 144 dental students | To measure students attitudes about societal expectations, health professionals responsibility, access to care and students efficacy to positively impact the need for expanded oral health of the underserved. | Attitudes towards care for the oral health of the underserved remained positive over the study period, but uncertainty increased as to who was responsible to fulfil the obligation. |
| Jessani, Athanasakos Kachwinva,2024. (42) | East Africa (Rwanda)/ Canada | Qualitative assessment of reflective essays. | 8 dental students. | Explores the experiences and impacts of a Global health community service-learning programme in East Africa among undergraduate dental learners from Canadian institute. | 5 Major themes emerged 1: Experiential clinical learning 2: Cultural humility and social awareness 3: awareness of contrasting healthcare systems 4: commitment to service 5: personal and professional growth. |
| Nandakumar, Robinson, 2011. (43) | England | Educational case study. | 1 dental student | To provide an example how dental public health can be taught to undergraduates.  Requirement for students to study patients in the context of their environment via their social history as part of an outreach project. | Demonstrates how outreach training can promote learning of the social determinants of health. Danger of stereotyping noted in that each patient should not be assumed to have the characteristics of the local population. |
| Cooper, Kim, Duderstadt, Et Al 2017 (44). | United states | Quantitative study  Evaluation study 10-week interprofessional practise and education course | 31 students | To evaluate changes in knowledge, confidence, attitude, and clinical practice in children’s oral health on completion of the course. | IPE Evaluation showed that offering an interprofessional course on children’s oral health to graduate students in dentistry, nursing and osteopathic medicine can improve their knowledge, confidence and practice towards children’s oral health and expand their professional goals to include caring for underserved, minority children. |
| Levesque, Dupere, Morin Et Al 2015 (45) | Canada | Summary report | NA | To describe and reflect on a multi-agency collaboration to produce an educational film on poverty- with the aim to demonstrate how participatory research can enhance knowledge, promote critical reflection, and address complexity. | Listening to others group produced a short 15min film depicting the social context and oral health care of a 37-year-old mum on welfare. Outlined structural causes of poverty and social exclusion, highlighting need for reduced prejudice within dentistry. |
| Lévesque, Levine, Bedos 2016. (46) | Canada | Qualitative case study. | 15 dental staff members. | Original onsite continuing education (CE) course on poverty was co-developed by researchers, dental professionals, and community organizations. Integrating patient narratives and a short film, course material aims to elicit critical reflection and provide coaching for practice improvements. Implications for research discussed. | Primary care practitioners are frequently unprepared to take into account the effects of social determinants on underprivileged patients' health and health management. |
| Levesque, Dupere, Loignon et Al 2009 (47) | United states | Qualitative study – Interviews with representatives from low-income backgrounds. | 6 Participants | Describes participative methods, the content of the Listening to each other DVD to improve interaction between underprivileged people and dental care providers. | Collaborating with anti-poverty group has beneficial effects.  Highlighted:  1: The importance of teeth and oral health and the stigma with loss of teeth vs advanced restorative care.  2: Relationship with oral health professionals- Importance of empathy from the whole team.  3: Barriers to accessing dental services.  4: Everyday life on welfare. |
| Ramos-Gomez, Silva, Law et Al 2014. (48) | United states | Quantitative  Cross sectional retrospective surveys. | 14 Paediatric dental residents | Outlines CHAT-PD programme (Community health and advocacy training, paediatric dentistry) to improve children’s health and the impact to the dental profession. | Outcomes measured- candidates were reported to have a greater perception of disease management and risk assessment tools, increased participation in policy and advocacy and increased understanding and acceptability of working with low-income families. |
| Brown, Apenteng, Opoku. 2022 (49) | United states | Cross sectional qualitative study-Online survey | 370 patients | Identify the factors associated with the occurrence of dental-patient provider cost conversations | Screening for financial hardship appeared to be the most important factor associated with cost conversations- Enables identification of financial stress and cost responsive, quality, patient centred oral healthcare to all patients. |
